# Clinical outcomes of radio‐hyperthermo‐chemotherapy for soft tissue sarcoma compared to a soft tissue sarcoma registry in Japan: a retrospective matched‐pair cohort study

**DOI:** 10.1002/cam4.1366

**Published:** 2018-02-26

**Authors:** Hisaki Aiba, Satoshi Yamada, Jun Mizutani, Norio Yamamoto, Hideki Okamoto, Katsuhiro Hayashi, Hiroaki Kimura, Akihiko Takeuchi, Shinji Miwa, Akira Kawai, Kenichi Yoshimura, Hiroyuki Tsuchiya, Takanobu Otsuka

**Affiliations:** ^1^ Department of Orthopedic Surgery Nagoya City University Graduate School of Medical Sciences Nagoya Aichi Japan; ^2^ Department of Orthopedic Surgery Graduate School of Medical Sciences Kanazawa University Kanazawa Ishikawa Japan; ^3^ Department of Musculoskeletal Oncology National Cancer Center Hospital Tokyo Japan; ^4^ Department of Biostatistics Innovative Clinical Research Center Kanazawa University Kanazawa Ishikawa Japan

**Keywords:** hyperthermia sarcoma, neoadjuvant chemotherapy, radiotherapy, soft tissue sarcoma, soft tissue sarcoma‐chemotherapy

## Abstract

Regional hyperthermia is considered to enhance the antitumor effects of chemotherapy and radiotherapy. In this study, we confirmed the efficacy of concomitant radiotherapy, hyperthermia, and chemotherapy (RHC) for neoadjuvant treatment of malignant soft tissue sarcoma (STS). From 1994 to 2013, we performed RHC in 150 patients. This study was limited to 60 patients using the following exclusion criteria: salvage for recurrence or unplanned excision, trunk location, metastasis at initiation, non‐STS, and no definitive surgery. As a control group, we collected data from 11,031 patients in the Bone and Soft Tissue Tumor Registry in Japan (BSTT). We performed multivariate logistic regression analysis, and propensity scores were created for comparisons between groups. The primary outcome of this study was to compare oncologic outcomes (5‐year local control rate [LC] and overall survival rate [OS]). In the RHC group, two local recurrences (3.3%) occurred, and no patients underwent amputation. Margins of definitive surgery were not identical between groups [wide margins (60.0% vs. 85.3%), marginal margins (28.3% vs. 10.5%), and intralesional margins (7.4% vs. 4.2%), RHC and BSTT groups, respectively, *P* < 0.001]. After adjustment, the difference in OS was not significant between groups (HR = 1.26, *P* = 0.532); however, a statistically significant difference in LC was observed (HR = 4.82, *P* = 0.037). RHC resulted in a high LC at 5 years compared to the BSTT group, and amputation was averted in the RHC group, despite the wider margins in the BSTT group. This indicates that less invasive surgery might be achieved with effective neoadjuvant therapy.

## Introduction

Soft tissue sarcoma (STS) is an extremely rare disease, with an annual incidence of three per 100,000 people, accounting for ≤1% of all malignant tumors 1. About 60–70% of STS lesions are located in the extremities 2. The primary goal of therapy for extremity STS is complete tumor resection with a negative margin. However, clinicians are sometimes distressed when making the choice between sacrificing neurovascular bundles and/or amputation and preserving function, especially for tumors located near critical areas for function or circulation.

Regional hyperthermia is considered to enhance the antitumor effects of chemotherapy and radiotherapy by compensating for the weak points of these therapies 3, 4, 5, 6, 7, 8. The prospective randomized multicenter EORTC (European Organisation of Research and Treatment of Cancer) 62,691 trial revealed that adding hyperthermia to chemotherapy (etoposide [VP‐16] + ifosfamide [IFO] + doxorubicin [ADR]; EIA) provided a benefit to patients with high‐risk STS 9. This trial enrolled patients with extremity STS (43.2%) and nonextremity STS (55.8%); 72.0% of patients underwent neoadjuvant therapy before their first resection, while 28.0% had undergone previous inadequate resection. The results showed that hyperthermia plus chemotherapy significantly increased local progression‐free survival (hazard ratio [HR] = 0.58, *P* = 0.003) and disease‐free survival (HR = 0.70, *P* = 0.011) compared to chemotherapy alone in both extremity and nonextremity STS. The surgical approaches did not differ between groups; they were almost identical (R0 resection, 53 and 42; R1 resection, 35 and 36; and R2 resection, 9 and 14 in the hyperthermia plus chemotherapy and chemotherapy alone groups, respectively), and similar numbers of patients required amputation (7 and 9 in the hyperthermia plus chemotherapy and chemotherapy alone groups, respectively).

In our institution, in addition to regional hyperthermia and chemotherapy, we simultaneously perform radiotherapy (radio‐hyperthermo‐chemotherapy [RHC]) as neoadjuvant therapy for high‐grade STS. In this study, we assessed the oncologic outcomes of patients treated with RHC, limiting patients to those receiving neoadjuvant therapy, having extremity STS, and undergoing first resection, by comparing the RHC group to a nationwide database in Japan (Bone and Soft Tissue Tumor Registry [BSST]) by propensity score matching. Furthermore, to assess the contribution of RHC to other neoadjuvant therapies (chemotherapy, radiotherapy, chemotherapy plus radiotherapy, or no neoadjuvant therapy), we compared oncologic outcome by extracting subgroups from whole BSTT group according to neoadjuvant therapy.

## Methods

### Patients

From 1994 to 2013, RHC was performed in 150 patients with STS. Eligible patients were 15–70 years of age and had Fédération Nationale des Centres de Lutte Contre le Cancer (FNCLCC) grade 2 or 3 extremity STS (low‐grade sarcomas, such as dermatofibrosarcoma protuberans or well‐differentiated liposarcoma, were not suitable for RHC). Tumors with diameters ≥5 cm were exclusively subjected to RHC, and tumors with diameters <5 cm with “tail signs 10” or thick fascial enhancement extending from the tumor margin (e.g., myxofibrosarcoma and undifferentiated pleiomorphic sarcoma [UPS]) were also included in the RHC group. Exclusion criteria were: salvage treatment for recurrence or unplanned excision, metastasis at study initiation, chondro‐osseous tumors, small round cell sarcoma, and no definitive surgery. All of the patients were fully informed about RHC therapy and potential associated adverse events and provided consent to participate. Anytime during the RHC procedure, patients had the latitude to change their treatment plans to alternative therapies. To minimize adverse events, patients were required to fulfill the following criteria: (1) white blood cell count, >3,000/*μ*L; (2) platelet count, >7.5 × 10^4^/*μ*L; (3) hemoglobin, >7.0 g/dL; (4) creatinine clearance, >60 mL/min; (5) normal hepatic function; and (6) ejection fraction (EF), >60%.

### RHC protocol

**Figure 1:**
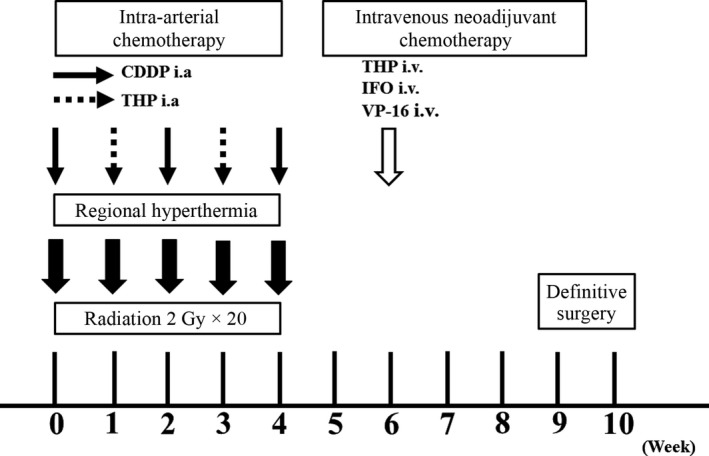
RHC protocol.

Prior to RHC therapy, angiography was performed to assess tumor blood flow, and a catheter was simultaneously inserted with a reservoir placed into the artery feeding the tumor (Fig. 1). Anticancer drugs were intra‐arterially injected through this reservoir. Radiotherapy was initiated on day 1, and 2 Gy per fraction were delivered 20 times (on days 1–5, 8–12, 15–19, and 22–26) for a total of 40 Gy. Following radiotherapy, thermotherapy was simultaneously initiated with intra‐arterial injection of anticancer drugs. Cisplatin (CDDP, 100 mg/m^2^) on days 1, 15, and 29 and pirarubicin (THP [a derivative of ADR], 30 mg/m^2^) on days 8 and 22 were alternatively administered. The treatment interval was extended depending on patient status, and, if necessary, colony‐stimulating factor was administered. Two weeks after RHC therapy was completed (day 43), additional neoadjuvant chemotherapy (IFO, 2 mg/m^2^; VP‐16, 100 mg/m^2^; and THP, 30 mg/m^2^; modified Rosen T‐16 regimen 11) was administered intravenously.

For thermotherapy, an 8‐MHz radiofrequency capacitive heating system (Thermotron RF‐8: Yamamoto VINITA, Osaka, Japan) was used. Tumor temperature was monitored by inserting a hyperthermia needle into the tumor and inserting a thermocouple thermometer (0.64 mm, Thermotron CE‐150, Yamamoto VINITA, Osaka, Japan) into the space. The treatment objective was to achieve a temperature of ≥42.5°C for 60 min (T42.5*60). Three categories were established based on the number of cycles in which T42.5*60 was achieved: poor hyperthermia, T42.5*60 was not achieved; mild hyperthermia, T42.5*60 was achieved in 1–3 cycles; and complete hyperthermia, T42.5*60 was achieved in 4–5 cycles 12.

During the RHC treatment period, the efficacy of RHC was carefully monitored using several modalities, including dynamic magnetic resonance imaging (MRI), computed tomography (CT), Tl^201^‐scintigraphy, and/or positron emission tomography (PET). If the response to RHC was a partial response (PR) or complete response (CR) in modified Response Evaluation Criteria in Solid Tumors (RECIST) or PET Response Criteria in Solid Tumors (PERCIST), marginal resection of the tumor was performed as minimally invasive surgery with preservation of neurovascular bundles.

### BSTT group

The BSTT is a nationwide organ‐specific cancer registry for bone and soft tissue tumors in Japan 13. The registry is funded by the Japanese Orthopaedic Association (JOA) and promoted by the National Cancer Center. A total of 89 JOA‐certified hospitals specializing in musculoskeletal oncology are obliged to participate in this registry, but participation of other hospitals is voluntary. Annual reports include patient characteristics including basic patient data (sex, age, date of diagnosis, and treatment status at first visit [initial therapy or not]), tumor data (diagnosis, histologic details [malignant or benign disease and histologic grade for malignant tumors], tumor location, and data required for TNM staging [seventh edition AJCC staging]), surgical data (date of definitive surgery, type of surgery, reconstruction details, and additional surgery for complications), and information about neoadjuvant/adjuvant treatment (including chemotherapy, radiotherapy, and/or hyperthermia).

A second survey collects information on prognosis at 2, 5, and 10 years after initial registration. This includes information about several outcomes at the time of latest follow‐up, such as local recurrence, distant metastasis, and oncologic outcome. The use of the BSTT database for the purpose of clinical research was initiated in 2014 after the approval of the Musculoskeletal Tumor Committee of the JOA.

### Statistical analysis

The primary goal of this study was to validate the efficacy of the RHC by investigating oncologic outcomes (local control rate, defined as the time from surgery to local recurrence, and overall survival, defined as the time from surgery to death due to any cause) by comparing RHC and BSTT database. Secondary outcomes were to investigate each contribution of neoadjuvant therapy by comparing extracted subgroups from whole BSTT group according to neoadjuvant therapy.

Statistical parameters obtained from the BSTT database and our hospital included age, sex, tumor location, tumor size, tumor histology, tumor depth, neoadjuvant therapy (chemotherapy and/or radiotherapy), and adjuvant therapy (chemotherapy and/or radiotherapy). We also included information about surgery, such as usage of prosthesis, surgical margins, requirement for plastic surgery, infection, and delayed skin healing. Multivariate logistic regression analysis was performed to determine associations between these factors and RHC. Propensity scores were calculated using a logistic regression model by including the weights of the contributions of each patient's demographic data, except for information about neoadjuvant or adjuvant therapy. This was because all patients in the RHC group underwent both neoadjuvant chemotherapy + radiotherapy and 70% of patients in the RHC group underwent adjuvant chemotherapy so that the information about neoadjuvant or adjuvant therapy could strongly affect the fair matching. After calculation of propensity scores, we matched patients in both groups according to propensity score with a nearest‐neighbor algorithm, allowing a maximum tolerated difference between propensity scores of ≤30% of the propensity score. Considering the differences in the number of patients in each group, 5:1 propensity score matching was used for the whole BSTT group vs RHC group. Standardized group differences were calculated by Kaplan–Meier analysis. For the analysis of the risk factors for oncologic outcomes, hazard ratios were calculated using a Cox proportional hazards model. All statistical analyses were conducted using SPSS version 24 (IBM^®^). *P*‐values <0.05 were considered statistically significant.

### Ethics approval

This retrospective study has received the approval of the local committee of Nagoya City University Hospital and has been conducted in compliance with the guidelines of the Helsinki Declaration of 1975. Also, use of the BSTT database was approved by the Institutional Review Board of the JOA.

## Results

**Figure 2:**
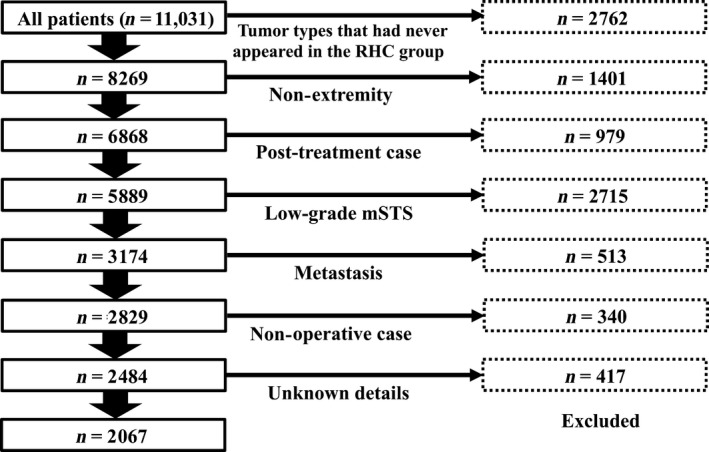
Patient flow.

From 2007 to 2013, 11,031 patients were registered in the BSTT database. Before matching, we excluded ineligible data from the database; 2,762 patients with some tumor types that had not coincidentally appeared in the RHC group, such as dedifferentiated liposarcoma and malignant peripheral nerve sheath tumor, were excluded. Next, 1,401 patients with nonextremity STS and 979 patients with unplanned excisions or recurrences were excluded; 2,715 patients with low‐grade sarcoma, such as well‐differentiated liposarcoma, solitary fibrous tumor, and low‐grade myofibroblastic sarcoma, were excluded; 513 patients with metastasis during the initial period and 340 patients who had not undergone any surgery for primary tumors were excluded; and 417 patients for whom information was lacking were excluded. In summary, a total of 2,067 patients were eligible for this study (whole BSTT group; Fig. 2).

**Figure 3:**
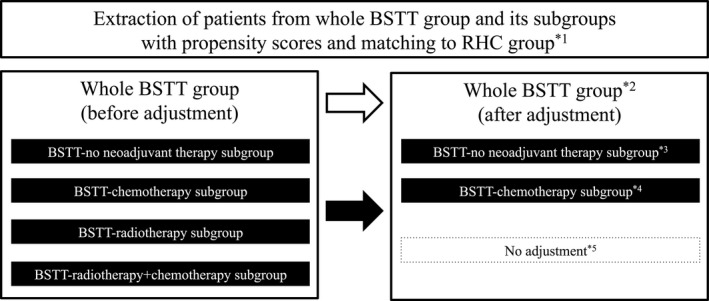
Extraction of patients from whole BSTT group and its subgroups with propensity scores and matching to RHC group. 
*1. Propensity scores were calculated using a logistic regression model by including the weights of the contributions of each patient's demographic data, except for information about neoadjuvant or adjuvant therapy.*2. 5:1 propensity score matching was used for the whole BSTT group versus RHC group.*3. 5:1 propensity score matching was used for the BSTT‐no neoadjuvant therapy subgroup versus RHC group.*4. 3:1 propensity score matching was used for the BSTT‐no neoadjuvant therapy subgroup versus RHC group.*5. The numbers of patients were insufficient for matching analysis, so no data adjustment was performed.

For the subgroup analysis, we assigned patients who underwent no neoadjuvant therapy, neoadjuvant chemotherapy, neoadjuvant radiotherapy, or neoadjuvant chemotherapy + radiotherapy into the BSTT‐no neoadjuvant therapy subgroup, the BSTT‐chemotherapy subgroup, the BSTT‐radiotherapy subgroup, and the BSTT‐chemotherapy + radiotherapy subgroup, respectively (Fig. 3).

In addition, patients in the RHC group were excluded for the following reasons: salvage therapy for recurrence or unplanned excision (*n* = 26); trunk location (*n* = 4); metastasis at study initiation (*n* = 11); non‐STS (*n* = 30); no definitive surgery (*n* = 8); and other (*n* = 11). Finally, 60 patients were included in the RHC group during the study period. An average of 4.58 cycles of RHC were given as neoadjuvant therapy. T42.5*60 was achieved in 68.3% of patients, and 48.3% of patients were considered to achieve complete hyperthermia (T42.5*60 = 4–5 cycles). However, 31.7% of patients achieved poor hyperthermia. Definitive surgery was performed with wide margins (60.0%), marginal margins (28.3%), and intralesional margins (11.7%). After surgery, based on patients status, the pathological analysis of margin, histological grade of tumor, or response to neoadjuvant therapy, 70.0% of patients received intravenous adjuvant chemotherapy with IFO, VP‐16, and THP (up to 5 courses). Histological analysis revealed that 21.7% of patients had a complete response (total tumor necrosis) and 40% of patients had >90% necrosis; however, 13% of patients had a poor response (<50% necrosis). The mean follow‐up period was 7.14 ± 4.84 years after definitive surgery.

In the RHC group, delayed skin healing after surgery (>2 weeks) occurred in 20 patients. Among these, eight patients required additional procedures (debridement, surgical skin closure, or skin flap), but all cases were managed. One case of femoral head necrosis occurred, for which total hip arthroplasty was performed. Moreover, one case of prolonged fever of unknown origin occurred.

### Whole BSTT group versus RHC group

**Figure 4:**
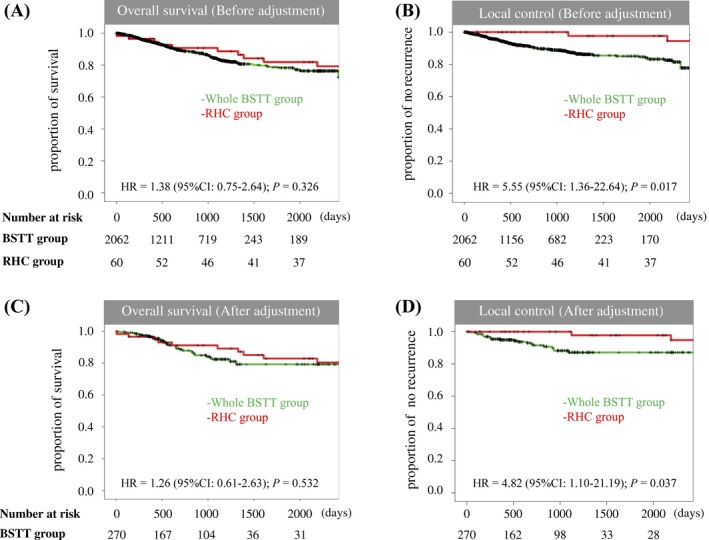
Whole BSTT group vs RHC groups. Kaplan–Meier curves for overall survival before adjustment (A) and time to local recurrence before adjustment (B). Kaplan–Meier curves for overall survival (C) and time to local recurrence (D), after adjustment in the whole BSTT group versus RHC groups.

First, the whole BSTT group was compared to the RHC group (Table 1). Marginal tumor resection was more frequently performed in the RHC group (28.3% vs. 10.5%, RHC group vs. whole BSTT group, respectively; *P* < 0.001, Table 2). The 5‐year overall survival rate was 78.3% and 81.2% in the whole BSTT and RHC groups, respectively (*P* = 0.326; Fig. 4A). A statistically significant difference was observed in 5‐year local control rates between the whole BSTT (85.1%) and RHC (97.7%) groups (*P* = 0.017, Fig. 4B).

**Table 1:** Patient characteristic in the Whole BSTT and RHC group

	Whole BSTT group (*n* = 2,066)			BSTT group after adjustment (*n* = 270)			RHC group (*n* = 60)	
Sex								
Male	1,121	54.2%	*P* = 0.057	159	58.9%	*P* = 0.265	40	66.7%
Female	945	45.7%		111	41.1%		20	33.3%
Histology								
UPS	721	34.9%	*P* = 0.582	92	34.1%	*P* = 0.531	22	36.6%
Liposarcoma (myxoid/round cell)	382	18.5%	*P* = 0.054	79	29.3%	*P* = 0.886	17	28.3%
Synovial sarcoma	196	9.5%	*P* = 0.063	55	20.4%	*P* = 0.514	10	16.7%
Leiomyosarcoma	237	11.5%	*P* = 0.017	9	3.3%	*P* = 0.495	1	1.7%
ASPS	16	0.8%	*P* < 0.001	10	3.7%	*P* = 0.641	3	5.0%
Rhabdomyosarcoma	61	3%	*P* = 0.559	10	3.7%	*P* = 0.890	2	3.4%
Myxofibrosarcoma	435	21.9%	*P* = 0.116	20	7.4%	*P* = 0.806	5	8.3%
Depth								
Superficial	481	23.3%	*P* = 0.071	30	11.1%	*P* = 0.844	8	13.3%
Deep	1,585	76.7%		240	88.9%		52	86.7%
Age, years								
Mean ± SD	61.73 ± 18.33		*P* < 0.001	49.72 ± 20.54		*P* = 0.400	47.61 ± 16.33	
Length of maximum diameter, cm								
Mean ± SD	9.57 ± 8.03^a^		*P* = 0.573	10.06 ± 9.29		*P* = 0.312	9.02 ± 4.47	
Position								
Shoulder	89	4.3%	*P* = 0.713	13	4.8%	*P* = 0.274	1	1.7%
Upper arm	170	8.2%	*P* = 0.021	23	8.5%	*P* = 0.065	1	1.7%
Elbow	39	1.9%	*P* = 0.901	5	1.9%	*P* = 0.923	1	1.7%
Forearm	106	5.1%	*P* = 0.532	16	5.9%	*P* = 0.424	2	3.4%
Hand	18	0.9%	*P* = 0.468	4	1.5%	*P* = 0.343	0	0%
Hip	163	7.9%	*P* = 0.900	25	9.3%	*P* = 0.822	5	8.3%
Thigh	1,044	50.5%	*P* = 0.051	126	46.7%	*P* = 0.020	38	63.3%
Knee	114	5.5%	*P* = 0.863	16	5.9%	*P* = 0.781	3	2.6%
Lower leg	277	13.4%	*P* = 0.721	37	13.7%	*P* = 0.793	9	15.0%
Foot	46	2.2%	*P* = 0.243	5	1.9%	*P* = 0.288	0	0%
Neoadjuvant chemotherapy								
Yes	395	19.1%	*P* < 0.001	85	31.5%	*P* < 0.001	60	100%
No	1,671	80.9%		185	68.5%		0	0%
Neoadjuvant radiotherapy								
Yes	104	5%	*P* < 0.001	22	8.1%	*P* < 0.001	60	100%
No	1,962	95%		248	91.9%		0	0%
Amputation								
Yes	137	6.6%	*P* = 0.039	15	5.6%	*P* = 0.062	0	0%
No	1,929	93.4%		255	94.4%		60	100%

ASPS, alveolar soft part sarcoma; UPS, undifferentiated pleiomorphic sarcoma.

Each *P*‐value was compared to RHC group.

a The numbers of patients with tumor under 5 cm in length of maximum diameter were 496 (24%), 65 (24%), and 12 (20%) (Whole BSTT group, BSTT group after adjustment, and RHC group, respectively).

**Table 2:** Local control rate and amputation rate by type of resection

Type of resection	Proportion				Local recurrence at 5 years		Amputation rate	
	Whole BSTT group		RHC group		Whole BSTT group	RHC group	Whole BSTT group	RHC group
Wide	1,763	85.3%	36	60.0%	7.8% (138/1763)	0% (0/36)	6.5% (115/1763)	0% (0/36)
Marginal	216	10.5%	17	28.3%	10.2% (22/216)	5.9% (1/17)	5.6% (12/216)	0% (0/17)
Intralesional	87	4.2%	7	11.7%	31.0% (27/87)	14.3% (1/7)	11.6% (10/87)	0% (0/7)

Negative prognostic factors for overall survival based on the results of multivariate analysis included histology (UPS, HR = 0.54 [95% CI: 0.38–0.77], *P* = 0.001; synovial sarcoma, HR = 0.45 [95% CI: 0.27–0.75], *P* = 0.002; leiomyosarcoma, HR = 0.39 [95% CI: 0.25–0.61], *P* < 0.001); sex (male<female, HR = 1.44 [95% CI: 1.11–1.88]); and tumor size (5–10 cm, HR = 0.44 [95% CI: 0.31–0.63], *P* < 0.001; 10–15 cm, HR = 0.40 [95% CI: 0.29–0.55], *P* < 0.001; >15 cm, HR = 0.23 [95% CI: 0.13–0.38], *P* < 0.001). Surgical margin (intralesional margin, HR = 0.53 [95% CI: 0.34–0.81], *P* = 0.004) was also a negative predictive factor.

For local recurrence at the surgical site, tumor size (5–10 cm, HR = 0.56 [95% CI: 0.37–0.84], *P* < 0.005; 10–15 cm, HR = 0.51 [95% CI: 0.35–0.75], *P* < 0.001; >15 cm, HR = 0.36 [95% CI: 0.21–0.60], *P* < 0.001) was identified as a negative prognostic factor by multivariate analysis. Surgical margin (intralesional margin, HR = 0.40 [95% CI: 0.23–0.71], *P* < 0.001) was also identified as a negative predictive factor. In contrast, myxoid liposarcoma was identified as a positive prognostic factor for local recurrence (HR = 2.87 [95% CI: 1.72–4.80], *P* < 0.001). Addition of hyperthermia was also determined to be a positive predictive factor (HR = 5.552 [95% CI: 1.36–22.64], *P* = 0.017).

**Figure 5:**
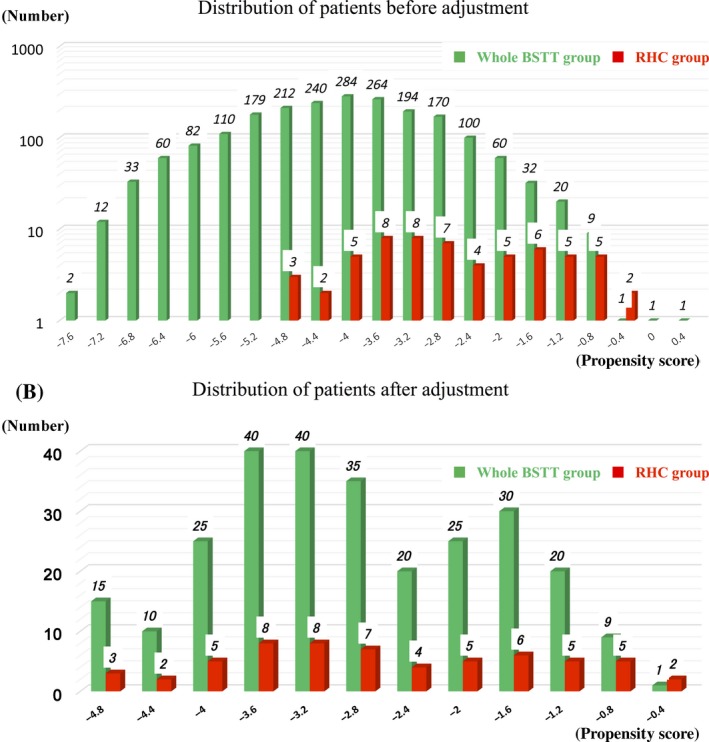
Distribution of patients in the whole BSTT and RHC groups. Green columns indicate the whole BSTT group, and red columns indicate the RHC group. Propensity scores were calculated by logistic regression by including weights of the contributions of each patients’ demographic data, except for information about neoadjuvant or adjuvant therapy and surgical data. (A) Before adjustment, (B) after adjustment.

Next, patient characteristics in the whole BSTT group were adjusted using propensity scores (5:1 matching). The distribution of patients before and after adjustment is shown in Figure 5A and B. The 5‐year overall survival rate in the BSTT group after adjustment was 79.3% (*P* = 0.532; Fig. 4C). A statistically significant difference (*P* = 0.041) in the local control rate was observed: The 5‐year local control rate in the BSTT group after adjustment was 87.1% (Fig. 4D).

### Subgroup analysis

#### BSTT‐chemotherapy subgroup versus RHC group

**Figure 6:**
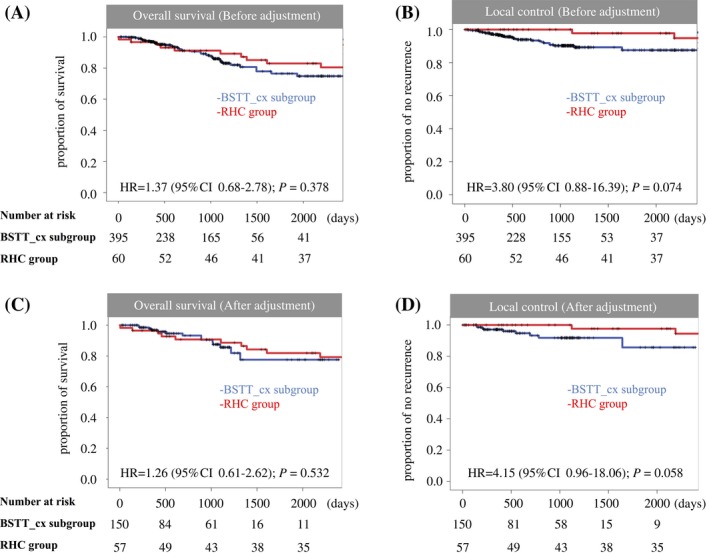
BSTT‐chemotherapy subgroup vs RHC group. Kaplan–Meier curves for overall survival (A) and time to local recurrence (B), before adjustment. Kaplan–Meier curves for overall survival (C) and time to local recurrence (D), after adjustment in the BSTT‐cx (neoadjuvant chemotherapy) subgroup versus RHC groups.

To clarify the contribution of neoadjuvant therapy, we extracted data from patients in the whole BSTT group who received neoadjuvant chemotherapy (Fig. 3). Neoadjuvant chemotherapy regimens were ADR + IFO (58%), mesna + ADR + IFO and dacarbazine (MAID, 11%), ADR + CDDP/IFO + VP‐16 (9%), ADR + CDDP (4%), and others (26%). Before adjustment, the 5‐year overall survival rate was 76.4% (Fig. 6A), and the 5‐year local control rate was 87.6% (Fig. 6B). No statistically significant difference in overall survival (*P* = 0.376) was observed; however, the local control rate was slightly superior in the RHC group (*P* = 0.074).

**Figure 7:**
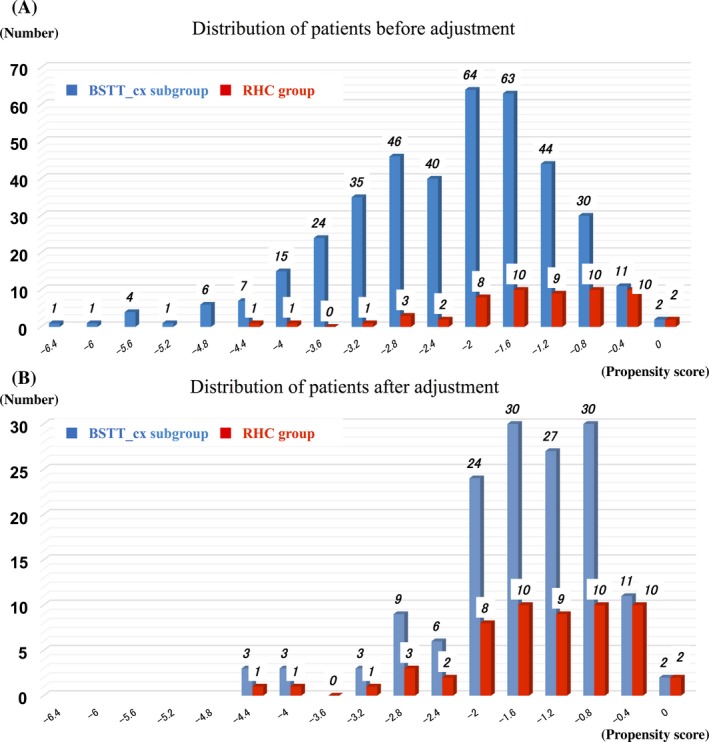
Distribution of patients in the BSTT‐chemotherapy subgroup and the RHC group. Blue columns indicate the BSTT‐chemotherapy subgroup (BSTT_cx subgroup), and red columns indicate the RHC group. Propensity scores were calculated by logistic regression by including weights of the contributions of each patient's demographic data, except for information about neoadjuvant or adjuvant therapy and surgical data. The ratio of BSTT_cx subgroup to RHC was therefore trimmed to 3:1. However, when matching pairs did not exist, the ratio was lenient. Also, there were no matching pairs for three patients (all with ASPS) in the RHC group, because none of the ASPS patients in the BSTT group received neoadjuvant therapy. (A) Before adjustment, (B) after adjustment.

The distribution of patients before adjustment is shown in Fig. 7A and B; scores were not homogeneous in the two groups. Next, patient characteristics in the BSTT‐chemotherapy subgroup were adjusted using propensity scores (3:1 matching). The characteristics before and after matching are shown in Table 3.

**Table 3:** Patient characteristics in the BSTT‐chemotherapy subgroup and the RHC group

	BSTT‐chemotherapy subgroup (*n* = 395)			BSTT‐chemotherapy subgroup after adjustment (*n* = 150)			RHC group (ASPS cases excluded)^a^ (*n* = 57)	
Sex								
Male	217	54.9%	*P* = 0.087	92	61.3%	*P* = 0.478	38	66.7%
Female	178	45.1%		58	38.7%		19	33.3%
Histology								
UPS	96	24.3%	*P* = 0.021	55	36.7%	*P* = 0.798	22	38.6%
Liposarcoma (myxoid/round cell)	89	22.5%	*P* = 0.321	47	31.3%	*P* = 0.834	17	29,8%
Synovial sarcoma	102	25.8%	*P* = 0.125	31	20.7%	*P* = 0.318	10	17.5%
Leiomyosarcoma	39	9.9%	*P* = 0.036	4	2.7%	*P* = 0.701	1	1.8%
ASPS	0	0%	*P* < 0.001	0	0%	n.a.	0	0%
Rhabdomyosarcoma	28	7.1%	*P* = 0.109	4	2.7%	*P* = 0.747	2	3.6%
Myxofibrosarcoma	41	10.4%	*P* = 0.624	9	6.0%	*P* = 0.478	5	8.8%
Depth								
Superficial	33	8.4%	*P* = 0.210	13	8.7%	*P* = 0.253	8	14.0%
Deep	362	91.6%		137	91.3%		49	86.0%
Age, years								
Mean ± SD	46.48 ± 17.03		*P* = 0.874	48.14 ± 15.47	0–94	*P* = 0.932	48.35 ± 15.63	15–70
Length of maximum diameter, cm								
Mean ± SD	11.1 ± 10.55^b^		*P* = 0.134	9.83 ± 4.90		*P* = 0.345	9.12 ± 4.37	
Position								
Shoulder	21	5.3%	*P* = 0.243	9	6.0%	*P* = 0.203	1	1.8%
Upper arm	20	5.1%	*P* = 0.267	9	6.0%	*P* = 0.203	1	1.8%
Elbow	10	2.5%	*P* = 0.728	6	4.0%	*P* = 0.425	1	1.8%
Forearm	25	6.3%	*P* = 0.401	6	3.3%	*P* = 0.867	2	3.5%
Hand	5	1.3%	*P* = 0.393	1	0.7%	*P* = 0.537	0	0%
Hip	35	8.9%	*P* = 0.982	10	6.7%	*P* = 0.602	5	8.8%
Thigh	185	46.8%	*P* = 0.097	82	70.1%	*P* = 0.382	35	61.4%
Knee	30	7.6%	*P* = 0.470	12	8.0%	*P* = 0.498	3	5.3%
Lower leg	54	13.7%	*P* = 0.666	14	9.3%	*P* = 0.187	9	15.8%
Foot	10	2.5%	*P* = 0.213	3	2.0%	*P* = 0.282	0	0%
Neoadjuvant radiotherapy								
Yes	44	11.1%	*P* < 0.001	20	13.3%	*P* < 0.001	57	100%
No	351	88.9%		130	86.7%		0	0%
Amputation								
Yes	33	8.4%	*P* = 0.023	14	9.3%	*P* = 0.017	0	0%
No	362	91.6%		136	90.7%		57	100%

ASPS, alveolar soft part sarcoma; UPS, undifferentiated pleiomorphic sarcoma.

Each *P*‐value was compared to RHC group.

a Three patients with ASPS in the RHC group were excluded because none of the ASPS patients in the BSTT group received neoadjuvant therapy.

b The numbers of patients with tumor under 5 cm in length of maximum diameter were 75 (19%), 25 (17%), and 11 (19%) (BSTT‐chemotherapy subgroup, BSTT‐chemotherapy subgroup after adjustment, and RHC group [ASPS cases excluded]), respectively).

After adjustment, overall survival at 5 years was 77.6%; the difference between the BSTT‐chemotherapy subgroup and RHC groups was not statistically significant (*P* = 0.532, Fig. 6C). With respect to local control rate, the rate at 5 years was 85.7%. No statistically significant difference in local control rate was observed (*P* = 0.058, Fig. 6D).

#### Comparison to other subgroups

**Figure 8:**
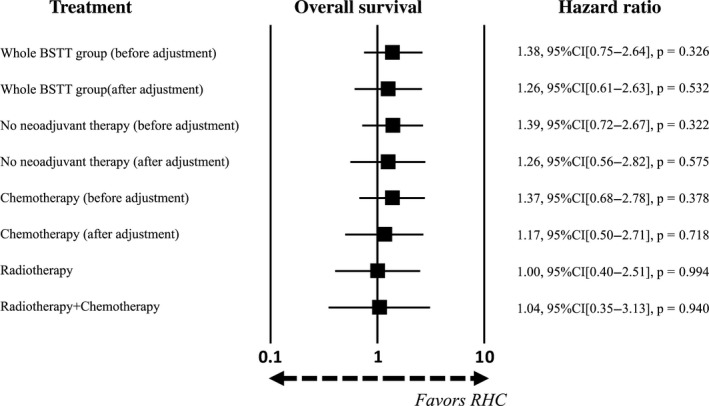
Contribution of neoadjuvant therapy to overall survival compared to the RHC group. Each subgroup was extracted from whole BSTT group and trimmed using propensity scores. Hazard ratio was calculated by comparing whole BSTT group and its subgroups with RHC group by log‐rank analysis.

**Figure 9:**
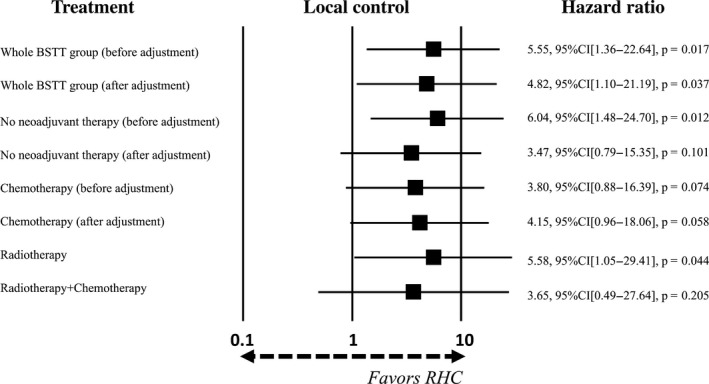
Contribution of neoadjuvant therapy to local control compared to the RHC group. Each subgroup was extracted from whole BSTT group and trimmed using propensity scores. Hazard ratio was calculated by comparing whole BSTT group and its subgroups with RHC group by log‐rank analysis.

Data from the no neoadjuvant therapy subgroup, radiotherapy subgroup, and radiotherapy + chemotherapy subgroup were extracted from the whole BSTT group to determine the contribution of each type of treatment on outcomes. The HRs between each subgroup and the RHC group were then calculated by log‐rank analysis. The patient characteristics in the no neoadjuvant therapy group (*n* = 1,611) were adjusted using a 5:1 matched‐pair analysis. Before adjustment, the HRs for overall survival rate and local control rate were 1.39 (95% CI: 0.72–2.67, *P* = 0.322) and 6.04 (95% CI: 1.48–24.74, *P* = 0.011), respectively. After adjustment, the HRs for overall survival rate and local control rate were 1.26 (95% CI: 0.56–2.82, *P* = 0.575) and 3.47 (95% CI: 0.79–15.35, *P* = 0.101), respectively. With respect to radiotherapy (*n* = 104) and radiotherapy plus chemotherapy (*n* = 44), the numbers of patients were insufficient for matching analysis, so no data adjustment was performed. The HRs for overall survival rate and local control rate were 1.00 (95% CI: 0.40–2.51, *P* = 0.994) and 5.58 (95% CI: 1.05–29.41, *P* = 0.044), respectively, in the radiotherapy group and were 1.04 (95% CI: 0.35–3.13, *P* = 0.940) and 3.65 (95% CI: 0.49–27.64, *P* = 0.205), respectively, in the radiotherapy plus chemotherapy group (Figs 8 and 9).

## Discussion

Hyperthermia refers to heating tumors, tissues, or systems to temperatures of up to 42°C, either to sensitize tissue to conventional treatments or to induce tumor regression 14. When combined with chemotherapy, hyperthermia is thought to enhance the antitumor activity of agents such as bleomycin 3, CDDP 4, and ADR 5, 6 by inhibiting their excretion and/or augmenting cancer cell sensitivity to these agents. In addition, both in vitro and in vivo studies have shown that hyperthermia has synergistic effects with radiotherapy. Hyperthermia compensates for the gaps in the cytotoxicity of radiotherapy by killing cells in S‐phase; these cells are generally resistant to radiotherapy, but susceptible to hyperthermia 7. Moreover, hypoxic cells, which are also impervious to radiotherapy, are vulnerable to hyperthermia because anaerobic metabolism creates a low‐pH environment around hypoxic cells, which enhances thermal effects 8. Our department has reported the direct effects of hyperthermia on STS in an in vitro/vivo study 15. According to previous reports, the synergistic effects are most pronounced when hyperthermia is simultaneously performed with radiotherapy and chemotherapy. On the basis of this observation, we established a protocol for performing hyperthermia, chemotherapy, and radiotherapy as a concomitant trimodal therapy for STS.

In 2010, Issels et al. reported the results of a randomized phase 3 trial of neoadjuvant chemotherapy for high‐grade STS, which provided considerable evidence supporting neoadjuvant treatment 9. These investigators concluded that local progression‐free survival and disease‐free survival were significantly prolonged by adding hyperthermia to EIA chemotherapy. In their study, the local control rate in the extremity STS subgroup was 92% at 2 years and 89% at 4 years. Nevertheless, while the survival outcomes in the chemotherapy and hyperthermia group were significantly better than those of the chemotherapy alone group, the recurrence rate was inferior to that observed in the present study. The lack of radiotherapy for 40% of patients and different inclusion criteria (Issels et al. included patients who underwent surgery before enrollment and those with non‐STS) could explain the differences between the results of the previous randomized study and those of the present study.

The results of a phase 3 randomized trial (the ISG‐GEIS trial) suggested the feasibility of neoadjuvant chemotherapy and radiotherapy allowing performance of less invasive surgery 16. The study authors revealed that combined preoperative chemotherapy and radiotherapy with positive surgical margins yielded a 100%‐local control. Likewise, in the present study, the local control rates of patients who underwent RHC were 100%, 94.1%, and 85.7% (wide resection, marginal resection, and intralesional resection, respectively), while the local control rates in the whole BSTT group were 92.2%, 89.8%, and 69.0% (wide resection, marginal resection, and intralesional resection, respectively). This stepwise decrease in the whole BSTT group suggests that tumor invasion around the surgical site and microresidual tumor tissue may be causes of recurrence. However, RHC might eradicate tumor progression around the surgical site, thereby improving the local control rate. Indeed, Matsumoto et al. reported the relationship between margins and the local control rate of infiltrative highly malignant soft tissue tumors in a relatively large population 17. The local control rates were 90%, 80%, 57%, and 30% (wide margins, inadequate wide margins, marginal resection, and intralesional resection, respectively). These results indicate that neoadjuvant therapy with RHC can downsize tumors, making it possible to achieve closer margins.

No amputations were performed in this study. This might have occurred due to a selective bias in the study inclusion criteria. However, in our experience, to achieve limb preservation, the application of amputation is limited for elderly patients (>70 years), patients with multiple recurrences, and patients with multiple metastases at initial diagnosis. These criteria might not differ from those of other hospitals in Japan. Actually, in our institution, patients who underwent amputation during this study period for STS included patients with multiple recurrences due to unplanned excision (*n* = 3), patients of advanced age (*n* = 2), and patients with multifocal lesions due to neurofibromatosis type 1 (*n* = 1). Moreover, if tumors invaded only major arteries, we preserved the arteries by marginal resection (if the tumor had dramatically shrunk) via reconstruction with an artificial, autograft, or in situ preparation technique 17. However, this selection bias might have affected the results even after compensation by propensity‐matching analysis. Moreover, the second limitation was that the collection of data from the BSTT database was not ideal. Details of neoadjuvant therapy, such as doses or amounts of chemotherapy administered, were unclear. Third, the relatively low number of patients in the RHC group was another limitation. The estimated one‐tailed power of this study for comparing the LC between RHC and whole BSTT group was almost 0.80. Due to the rarity of highly malignant STS and the time‐consuming nature of RHC, it was difficult to accrue an appropriate number of patients in this study. Moreover, the RHC procedure is challenging, and the installation of the device for heating tumors is expensive, so dissemination of this procedure on a nationwide basis will be somewhat difficult. Despite these limitations, we have demonstrated the possibility of limb‐sparing surgery for malignant STS involving reduction in surgical margins to preserve function.

In conclusion, RHC achieved a high 5‐year local control rate compared to an adjusted nationwide population group. Furthermore, amputation was averted in all patients. These results indicate that less invasive surgery might be achieved by high‐efficacy neoadjuvant therapy.

## Conflict of interest

None declared.
